# Neuroprotective effects of edaravone-administration on 6-OHDA-treated dopaminergic neurons

**DOI:** 10.1186/1471-2202-9-75

**Published:** 2008-08-01

**Authors:** Wen Ji Yuan, Takao Yasuhara, Tetsuro Shingo, Kenichiro Muraoka, Takashi Agari, Masahiro Kameda, Takashi Uozumi, Naoki Tajiri, Takamasa Morimoto, Meng Jing, Tanefumi Baba, Feifei Wang, Hanbai Leung, Toshihiro Matsui, Yasuyuki Miyoshi, Isao Date

**Affiliations:** 1 Department of Neurological Surgery, Okayama University Graduate School of Medicine, Dentistry and Pharmaceutical Sciences, Japan

## Abstract

**Background:**

Parkinson's disease (PD) is a neurological disorder characterized by the degeneration of nigrostriatal dopaminergic systems. Free radicals induced by oxidative stress are involved in the mechanisms of cell death in PD. This study clarifies the neuroprotective effects of edaravone (MCI-186, 3-methyl-1-phenyl-2-pyrazolin-5-one), which has already been used for the treatment of cerebral ischemia in Japan, on TH-positive dopaminergic neurons using PD model both *in vitro *and *in vivo*. 6-hydroxydopamine (6-OHDA), a neurotoxin for dopaminergic neurons, was added to cultured dopaminergic neurons derived from murine embryonal ventral mesencephalon with subsequet administration of edaravone or saline. The number of surviving TH-positive neurons and the degree of cell damage induced by free radicals were analyzed. In parallel, edaravone or saline was intravenously administered for PD model of rats receiving intrastriatal 6-OHDA lesion with subsequent behavioral and histological analyses.

**Results:**

*In vitro *study showed that edaravone significantly ameliorated the survival of TH-positive neurons in a dose-responsive manner. The number of apoptotic cells and HEt-positive cells significantly decreased, thus indicating that the neuroprotective effects of edaravone might be mediated by anti-apoptotic effects through the suppression of free radicals by edaravone. *In vivo *study demonstrated that edaravone-administration at 30 minutes after 6-OHDA lesion reduced the number of amphetamine-induced rotations significantly than edaravone-administration at 24 hours. Tyrosine hydroxylase (TH) staining of the striatum and substantia nigra pars compacta revealed that edaravone might exert neuroprotective effects on nigrostriatal dopaminergic systems. The neuroprotective effects were prominent when edaravone was administered early and in high concentration. TUNEL, HEt and Iba-1 staining *in vivo *might demonstrate the involvement of anti-apoptotic, anti-oxidative and anti-inflammatory effects of edaravone-administration.

**Conclusion:**

Edaravone exerts neuroprotective effects on PD model both *in vitro and in vivo*. The underlying mechanisms might be involved in the anti-apoptotic effects, anti-oxidative effects, and/or anti-inflammatory effects of edaravone. Edaravone might be a hopeful therapeutic option for PD, although the high therapeutic dosage remains to be solved for the clinical application.

## Background

Parkinson's disease (PD) is a neurodegenerative disorder characterized by slowly progressive degeneration of DA neurons in the substantia nigra pars compacta, with subsequent damage of nerve terminals accompanied by dopamine (DA) depletion in the striatum [1]. Although the neuropathological hallmarks of PD are well described, the etiology remains still undefined. However, accumulative evidences revealed many biochemical processes and molecular mechanisms as mediators of neuronal cell death in PD. Notably oxidative stress and mitochondrial dysfunction might be an important pillar of pathogenesis of PD [2].

6-hydroxydopamine (6-OHDA) is widely used for experimental models of PD [3]. It damages cells with dopaminergic neuronal attribute, including human neuroblastoma SH-SY5Y [4], PC12 cells derived from rat pheochromocytoma [5] and rat ventral mesencephalic neurons [6]. Furthermore, it is also a specific neurotoxin for DA neurons *in vivo *[2,7]. Intracellular lipids, proteins or DNA are damaged with consequent impairment of cell function induced by 6-OHDA. Mitochondrial oxidative phosphorylation with subsequent energy deprivation and excrement of 6-OHDA-auto-oxidation, including quinones and hydrogen peroxide (H_2_O_2_) are deeply involved in the cytotoxic processes [8]. As above described, mitochondrial dysfunction and oxidative stress might play important roles in the pathogenesis of PD [2], thus indicating that the experimental model using 6-OHDA might have essential mechanisms in common with PD. Furthermore, anti-oxidant agents, such as catalase, vitamin E, N-acetyl cysteine, ascorbic acid and pyruvate might exert neuroprotection for 6-OHDA-treated DA neurons [9].

Edaravone (3-methyl-1-phenyl-2-pyrazolin-5-one) is a potent scavenger of hydroxyl radicals, and is useful for patients suffering from ischemic stroke [10,11], with the involvement of peroxidation leading to neuronal cell death [12]. Neuroprotective effects of edaravone are explored using head trauma [13] and spinal cord ischemia [14]. Recent study demonstrated that edaravone suppress the production of nitric oxide and reactive oxygen species by activated microglia [15]. In both cerebral ischemia and PD, free radicals might be one of the critical pathogenesis which accelerates progression of disease. These results suggest that edaravone might have neuroprotective effects on 6-OHDA-treated DA neurons and might on slowly degenerated DA neurons in PD patients through anti-oxidative mechanisms.

In this study, first we explored the neuroprotective effects of edaravone on 6-OHDA-induced toxicity against murine ventral mesencephalic (VM) cell cultures and the underlying mechanisms. After confirming the effects *in vitro*, edaravone was intravenously administered to 6-OHDA-lesioned PD model of rats and evaluated behaviorally and immunohistochemically.

## Methods

### *In vitro *model of Parkinson's disease

#### Cell preparation

Murine DA neurons were cultured as described previously with minor modifications [16]. Tissue blocks of the ventral mesencephalon containing DA neurons were dissected from murine embryo (C57/B6) on day 14 of gestation after cervical dislocation with consequent trituration into single cell suspension. Cells were plated in mixed hormone MEM (MHM) supplemented with 1% fetal bovine serum at a density of 1 × 10^5 ^cells/well on poly-D-ornithine and fibronectin-coated glass slides in 24-well plates (Nunc, Frankfurt, FRG). Cultures were maintained at 37 degrees C in an atmosphere of 5% CO_2 _plus 95% air and with 100% relative humidity. Forty-eight hours after initial plating, the medium was exchanged and the cells were used for the further experiments. The average number of mesencephalic neurons was 0.42 ± 0.07 × 10^5 ^cells/well at the beginning of the experiment with TH-immunoreactivity in 37 ± 12% of total cells.

#### Administration of 6-OHDA and edaravone

Edaravone (MCI-186, 3-methyl-1-phenyl-2-pyrazolin-5-one) was kindly provided from Mitsubishi Pharma (Japan). It was dissolved in 0.5 ml of 1 N NaOH and 8 ml of distilled water, and adjusted to pH 7 by addition of 1 N HCl. At 48 hours after the initial plating, the cultured cells were exposed to 40 μM 6-OHDA (Sigma) or PBS for 30 minutes, and then added 10^-6^, 10^-5^, 10^-4 ^or 10^-3 ^M edaravone, or saline as a control at 37 degrees C. The cells were incubated for 18 hours and developed to immunocytochemical investigations.

#### Immunocytochemistry

Cells were fixed with 4% paraformaldehyde (PFA) for 30 minutes and then washed three times for 5 minutes in PBS. They were incubated overnight at 4 degrees C with an antibody directed against tyrosine hydroxylase (TH, rabbit polyclonal IgG, 1: 500, Chemicon) with 10% normal goat serum (Vector). After several rinses in PBS, cells were incubated at room temperature for 30 minutes in sheep anti-rabbit IgG FITC conjugate (1: 500, Sigma) and 4',6-diamidino-2-phenylindole, dilactate (DAPI, 1: 500, Molecular Probes). The cells were then washed three times in PBS and mounted on albumin-coated slides and embedded with cover glass. After photographically captured, immunoreactive neurons were counted per high power field view selected at random (n = 3 in each well, 10,000 μm^2^). Six wells were assigned to each group for statistical analyses. Control studies involved exclusion of primary antibody substituted with 10% normal goat serum in PBS. No immunoreactivity was observed in these controls.

#### TUNEL staining and HEt staining

In order to explore the involvement of apoptosis in this study, a modified method for terminal deoxynucleotidyl transferase-mediated biotinylated UTP nick end labeling (TUNEL, Roche) and DAPI staining was also used. After edaravone (10^-6^-10^-3 ^M) or saline were administered into separate series of 6-OHDA-treated DA neurons, cells were fixed at 18 hours as described in the previous section. TUNEL staining was performed according to the manufacturer's instruction.

In order to detect the early production of superoxide anions after 6-OHDA addition, hydroethidine (HEt), selectively oxidized to ehidium by superoxide anions, was used. HEt (1 mg/ml in PBS) was administered to 6-OHDA-treated DA neuronal cell culture at 0, 10, 20 or 30 minutes after edaravone- or saline- administration. After 5-minute incubation with HEt, the cells were washed 3 times in PBS, fixed with PFA, washed with PBS containing DAPI and finally embedded with cover glass. The cells were observed using a fluorescent microscope at an excitation of 355 nm and an emission of 450 nm for HEt and stained cells were counted as described above [17].

### *In vivo *model of Parkinson's disease

#### Subjects

We used adult female Sprague-Dawley rats (Charles River, Japan) weighing 250–300 g at the beginning of the experiment, according to approved guidelines of the institutional animal care and use committee of Okayama University. They were housed two per cage in a temperature and humidity controlled room, maintained on a 12-hour light/dark cycle, and they had free access to food and water.

#### Surgical procedures

Seventy eight rats were deeply anesthetized with sodium pentobarbital (30 mg/kg, i.p.) and placed in a stereotaxic instrument (Narishige, Japan). After pre-treatment of desipramine (25 mg/kg, i,p., Sigma), 20 μg of 6-OHDA (4 μl of 5 μg/μl dissolved in saline containing 0.2 mg/ml ascorbic acid; Sigma) was injected into the right striatum with a 28-gauge Hamilton syringe into the following coordinates: 1.0 mm anterior to the bregma, 3.0 mm lateral to the sagittal suture, and 5.0 mm ventral to the surface of the brain with tooth-bar set at 0 mm [18]. The injection rate was 1 μl/minute, and the syringe was left in place for an additional 5 minutes before being retracted slowly (1 mm/minute). At 30 minutes or 24 hours after 6-OHDA lesion, 30, 100, or 250 mg/kg of edaravone or saline (2 ml) were intravenously administered slowly from the right femoral vein.

#### Behavioral testing

All rats were tested with amphetamine (2.5 mg/kg, Dainippon-Seiyaku, Japan) at 1 and 2 weeks after 6-OHDA lesion, and rotational behaviors were assessed for 60 minutes with a video camera. Full 360 degrees turns in the direction ipsilateral to the lesion were counted.

#### Fixation and Sectioning

At 2 weeks after 6-OHDA lesion, rats were deeply anesthetized with sodium pentobarbital (100 mg/kg), perfused from the ascending aorta with 200 ml of cold PBS, followed by 100 ml of 4% PFA in PBS. Brains were removed and post-fixed in the same fixative for 2 days followed by 30% sucrose in phosphate buffer (PB) until to be sunk completely. Six series of coronal sections were cut at a thickness of 40 μm with a freezing microtome and stored at -20 degrees C.

#### Immunohistochemistry

Free floating sections for TH immunohistochemistry were blocked by 0.3% hydroxygen peroxide in methanol for 3 minutes with subsequent incubation in 1.5% normal goat serum (Vector). Sections were then incubated overnight at 4 degrees C with rabbit anti-TH (1: 1,000; Chemicon) antibody with 10% normal goat serum. After several rinses in PBS, sections were incubated for 30 minutes in biotinylated donkey anti-rabbit IgG (1: 1,000, Jackson) then for 30 minutes in avidin-biotin-peroxidase complex (1: 200, Vector). Subsequently the sections were treated with 3, 4-diaminobenzidine (DAB, Sigma) and hydroxygen peroxide, mounted on albumin-coated slides and embedded with cover glass.

TUNEL and HEt staining were also performed to investigate the involvement of anti-apoptotic effects and radical scavenging activity of edaravone using 14 rats receiving saline or 250 mg/kg of edaravone-administration at 30 minutes after 6-OHDA lesioning and sacrificed at 5 days after 6-OHDA lesioning. Furthermore, in order to reveal the effects of edaravone on the inflammation induced by 6-OHDA-administration, immunofluorescent Iba-1 staining was also performed. Rabbit anti-Iba-1 antibody (1: 100, Wako Pure Chemical Industries, Osaka, Japan) was used as the primary antibody and Alexa Fluor 594 (Molecular Probes) as the secondary antibody.

### Morphological analysis

The density of TH-positive fibers and Iba-1-positive microglia in the striatum of rats receiving edaravone- or saline-infusion was determined and analyzed as described previously with a computerized analysis system (Olympus Sp-1000, Japan) [16,19] using 3 serial coronal section at the bregma level. Two areas adjacent to the needle tract of lesioned side and symmetrical contralateral side were analyzed, respectively. For counting the number of TH-positive neurons, every fifth 40 μm-thick coronal tissue section through the substantia nigra pars compacta (SNc) was explored using 3 coronal sections respectively at -4.8 and -5.3 mm to the bregma. The number of TH-positive cell bodies in the SNc was counted and used for the statistical analyses.

### Statistical Analysis

The data obtained were evaluated statistically using analysis of variance (ANOVA) and subsequent post hoc Scheffe's *F*-test or Mann-Whitney's U test. Statistical significance was preset at p < 0.05.

## Results

### Edaravone promotes the survival of DA neurons *in vitro*

**Figure 1:**
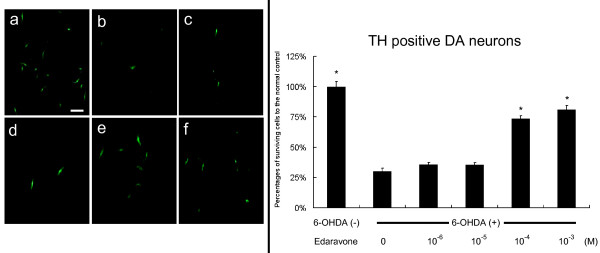
**Neuroprotective effects of edaravone on 6-OHDA-treated DA neurons *in vitro***. Left column: TH staining of DA neurons (a: non-6-OHDA-treated DA neurons) demonstrates that the number of surviving 6-OHDA-treated DA neurons significantly increased by the treatment with 10^-4 ^and 10^-3 ^M edaravone (e and f), compared to that with 10^-6 ^and 10^-5 ^M (c and d) edaravone as well as control without edaravone-administration (b). Scale bar: 30 μm. Right column: The graph demonstrates the number of surviving DA neurons by edaravone-administration. Data are shown as mean percentages of the cell number relative to the number of DA neurons without 6-OHDA-treatment +S.E. *p < 0.01 vs. 6-OHDA-treated DA neurons without edaravone-administration and those with low dose edaravone (10^-6 ^and 10^-5 ^M) by ANOVA.

We began our investigations into the neuroprotective capacity of edaravone on 6-OHDA-treated DA neurons *in vitro*. Exposure of 40 μM 6-OHDA resulted in a significant loss of TH-positive neurons to 30.2 ± 2.5% relative to the unexposed control (Fig. 1). Edaravone-administration (10^-4 ^and 10^-3 ^M) significantly reduced the loss of DA neurons induced by 6-OHDA (81.1 ± 3.5 and 73.6 ± 2.4%), compared to the 6-OHDA-treated DA neurons with 0, 10^-6 ^and 10^-5 ^M edaravone, although 10^-6 ^and 10^-5 ^M edaravone did not exert significant reduction of the cell loss (35.4 ± 1.9 and 35.8 ± 1.7%, One way ANOVA, F_5, 102 _= 125, p < 0.0001, Fig. 1).

**Figure 2:**
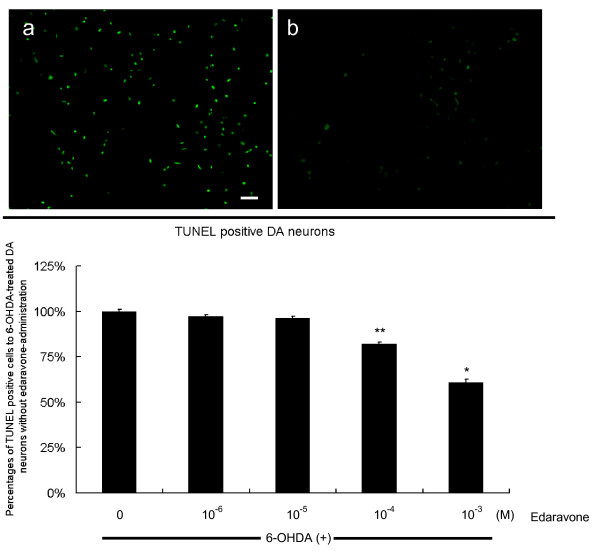
**Reduced TUNEL-positive apoptotic 6-OHDA-treated DA neurons with edaravone-administration *in vitro***. Upper column: TUNEL-positive 6-OHDA-treated DA neurons with 10^-3 ^M edaravone (b) significantly decreased, compared to those without edaravone-administration (a). Scale bar: 60 μm. Lower column: The graph demonstrates that TUNEL-positive 6-OHDA-treated DA neurons decreased by 10^-4 ^and 10^-3 ^M edaravone-administration. Data are shown as mean percentages of the cell number relative to the 6-OHDA-treated DA neurons without edaravone-administration +S.E. *p < 0.01 vs. 6-OHDA-treated DA neurons without edaravone-administration and those with low dose edaravone (10^-6 ^and 10^-5 ^M) by ANOVA. **p < 0.01 vs. 6-OHDA-treated DA neurons without edaravone-administration.

In order to determine that edaravone suppressed cell death through apoptosis, DA neurons were exposed to 40 μM 6-OHDA and then added 10^-6^, 10^-5^, 10^-4 ^or 10^-3 ^M edaravone with subsequent counting the number of swelling apoptotic cells exhibiting agglutinated and fragmented TUNEL-positive nuclei. Edaravone treatment (10^-3 ^M) significantly reduced the number of TUNEL-positive apoptotic cells to 60.9 ± 1.7 and 82.1 ± 0.8% relative to that of 6-OHDA-treated DA neurons without edaravone treatment, although 10^-6 ^and 10^-5 ^M did not suppress apoptosis (96.2 ± 0.7 and 97.3 ± 0.8%). 10^-4 ^M edaravone significantly suppressed apoptosis of DA neurons, compared to 6-OHDA-treated DA neurons without edaravone treatment (One way ANOVA, F_4, 45 _= 48, p < 0.0001, Fig. 2).

**Figure 3:**
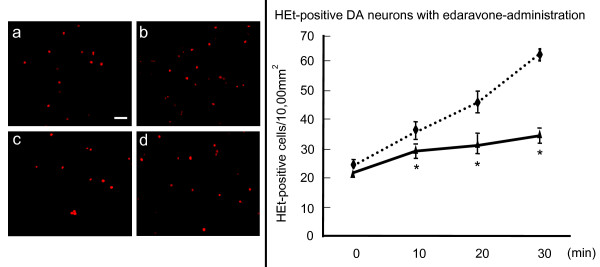
**Reduced number of HEt-positive cells by edaravone-treatment *in vitro***. Left column: Edaravone treatment (10^-3 ^M) significantly reduced the number of HEt-positive cells at 10, 20, 30 minutes after edaravone-administration, compared to the untreated 6-OHDA-exposed cells. (untreated 6-OHDA-treated DA neurons at 0 and 30 minutes (a and b); 6-OHDA-treated DA neurons with 10^-3 ^M edaravone at 0 and 30 minutes (c and d). Scale bar: 30 μm. Right column: The graph demonstrates that HEt-positive cells significantly decreased by edaravone-administration. Data are shown as the mean cell number ± S.E. Dotted line: HEt-positive cells without edaravone, Full line: HEt-positive cells with 10^-3 ^M edaravone. *p < 0.05 vs. 6-OHDA-treated DA neurons without edaravone-administration by ANOVA.

In order to demonstrate the production of superoxide anions, HEt staining was performed. Edaravone treatment (10^-3 ^M) significantly reduced the number of HEt-positive cells (21.5 ± 0.9, 29 ± 1.1, 31 ± 2.0, and 34 ± 1.7 cells/10,000 μm^2 ^at 0, 10, 20, and 30 minutes after edaravone administration), compared to the untreated 6-OHDA-exposed cells (24.3 ± 1.7, 35.7 ± 1.7, 45.7 ± 3.3, and 62.5 ± 0.9 cells/10,000 μm^2 ^at 0, 10, 20, and 30 minutes, Repeated Measures of ANOVA, F_3, 18 _= 21, p < 0.0001 and posthoc t-tests of p's < 0.01 for 10, 20, and 30 minutes after edaravone-administration, Fig. 3)

### Behavioral analyses *in vivo*

**Figure 4:**
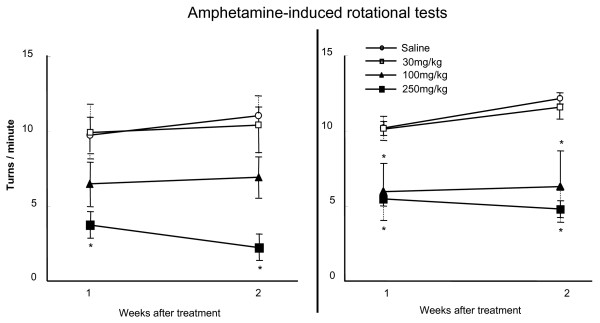
**Edaravone ameliorated the amphetamine-induced rotational behavior of PD model of rats**. Left graph: Rats receiving 250 mg/kg of edaravone infusion at 30 minutes after 6-OHDA lesion showed a significant reduction of the rotational number, although 30 and 100 mg/kg of edaravone did not exert significant effects. Data are shown as the mean rotational number per minute ± S.E. *p < 0.05 vs. rats receiving 30 mg/kg of edaravone and those without edaravone-administration. Right column: Edaravone administration (250 and 100 mg/kg) at 24 hours after 6-OHDA lesion significantly suppressed the rotational behavior, compared to rats receiving saline. Data are shown as the mean rotational number per minute ± S.E. *p < 0.05 vs. rats receiving 30 mg/kg of edaravone and those without edaravone-administration.

Next, we proceeded to the *in vivo *study using PD model of rats. There were no significant changes in the spontaneous behavior of rats receiving edaravone-administration at 30 minutes after 6-OHDA lesion (30 mg/kg: determined by the dose for ischemic stroke) or saline (data not shown). As shown in Fig. 4, in PD model of rats receiving intravenous saline-infusion, the number of amphetamine-induced rotations increased over time at 1 and 2 weeks (9.8 ± 1.1 and 11.1 ± 0.6 turns/hour). However, rats receiving 250 mg/kg of edaravone-administration at 30 minutes after 6-OHDA lesion showed a significant reduction of the rotational number (3.8 ± 0.9 and 2.3 ± 0.7 turns/hour at 1 and 2 weeks), although 30 and 100 mg/kg of edaravone did not exert significant effects (30 mg/kg: 9.9 ± 1.8 and 10.4 ± 1.9 turns/hour, 100 mg/kg: 6.5 ± 1.5 and 7.0 ± 1.4 turns/hour at 1 and 2 weeks, Repeated Measures of ANOVA, F_3, 24 _= 8.8, p < 0.0001 and posthoc t-tests of p's < 0.01 for both time periods, Fig. 4).

After confirmation of neuroprotective effects of intravenous administration of edaravone (250 mg/kg) at 30 minutes after 6-OHDA lesion on 6-OHDA-treated rats behaviorally, edaravone-administration at 24 hours were explored. Edaravone-administration (250 and 100 mg/kg) at 24 hours after 6-OHDA lesion significantly suppressed the rotational behavior (250 mg/kg: 5.5 ± 0.4 and 4.3 ± 0.6 turns/hour, 100 mg/kg: 6.0 ± 1.9 and 6.3 ± 2.4 turns/hour at 1 and 2 weeks), compared to rats receiving saline (10.2 ± 0.8 and 12.2 ± 0.4 turns/hour at 1 and 2 weeks, Repeated Measures of ANOVA, F_3, 22 _= 9.0, p = 0.0005 and posthoc t-tests of p's < 0.01 for both time periods, Fig. 4). Edaravone-administration (250 mg/kg) at 30 minutes significantly ameliorated rotational behavior, compared to that at 24 hours after 6-OHDA lesion (Repeated Measures of ANOVA, F_1, 12 _= 4.5, p = 0.04 and posthoc t-tests of p's = 0.04 for both time periods). Thus, edaravone significantly ameliorated the rotational behavior when it was administered earlier and in higher concentration.

### TH immunohistochemistry in the striatum and the SNc

**Figure 5:**
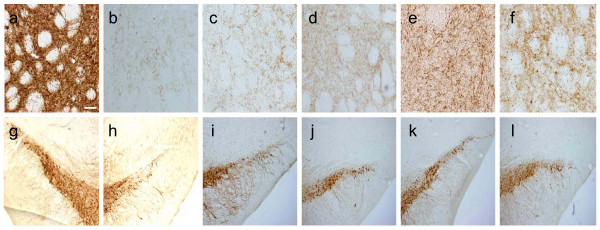
**Preserved TH-positive fibers in the striatum and neurons in the SNc of rats receiving edaravone-administration**. Photomicrographs demonstrate that 100 and 250 mg/kg of edaravone preserved TH immunoreactivity in the striatum and SNc (edaravone-administration at 30 minutes after 6-OHDA lesion, 30 mg/kg: c and i, 100 mg/kg: d and J, 250 mg/kg: e and k, edaravone administration at 24 hours after 6-OHDA lesion, 250 mg/kg: f and l), compared to the untreated 6-OHDA-lesioned rats (b and h). TH staining of the intact side of the striatum and SNc: a and h, TH staining of the striatum: a-f, and the SNc: g-l. Scale bar: 120 μm in a-f, 480 μm in g-l.

**Figure 6:**
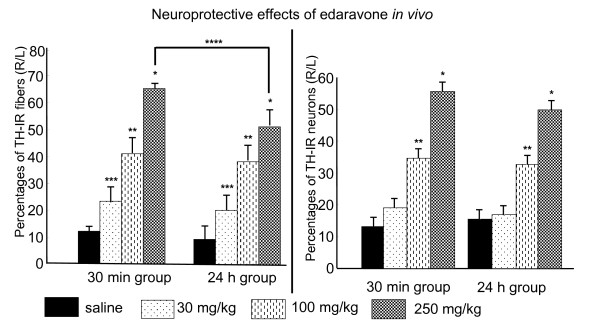
**Edaravone-administration exerted neuroprotective effects in a dose-responsive manner immunohistochemically *in vivo***. Left graph: TH staining of the striatum demonstrates neuroprotective effects of edaravone on 6-OHDA-lesioned striatal DA fibers in a dose-responsive manner. Data are shown as the percentages of TH-positive fibers relative to the intact side ± S.E. *p < 0.05 vs. rats in all other groups, **p < 0.05 vs. rats receiving 30 mg/kg of edaravone and those without edaravone-administration, ***p < 0.05 vs. rats without edaravone-administration, ****p < 0.05 (rats receiving edaravone at 30 minutes after 6-OHDA lesion (30 min group) vs. rats with edaravone at 24 hours (24 h group)). Right graph: TH staining of the SNc demonstrates neuroprotective effects of edaravone on 6-OHDA-lesioned striatal DA fibers in a dose-responsive manner. Data are shown as the percentages of TH-positive neurons relative to the intact side ± S.E. *p < 0.05 vs. rats in all other groups, **p < 0.05 vs. rats receiving 30 mg/kg of edaravone and those without edaravone-administration.

At 2 weeks after edaravone-()administration, TH staining was performed to evaluate the preserved DA fibers in the striatum and DA neurons in the SNc (Fig. 5). The density of TH-positive fibers in the 6-OHDA-lesioned striatum was compared with the contralateral side using a modified method of computerized image analysis system [19]. The preservation of TH-positive fibers in the striatum of rats receiving edaravone was significantly greater (30 minutes: 23.3 ± 1.5, 41 ± 1.2 and 65.2 ± 1.6%; 24 hours: 20.2 ± 0.9, 38.2 ± 1.7 and 51.4 ± 1.1% relative to the intact side at the dose of 30, 100 and 250 mg/kg, respectively) than those receiving the saline (30 minutes: 12.1 ± 0.5, 24 hours: 8.9 ± 0.3%, Repeated Measures of ANOVA, F_3, 16 _= 425, p < 0.0001 and posthoc t-tests of p's < 0.01 for all groups, Fig. 6).

The number of TH-positive neurons in the ipsilateral SNc of rats was analyzed as percentages relative to the number of counted DA neurons in the intact side. The preservation of TH-positive neurons in the SNc of rats receiving edaravone (100 and 250 mg/kg) was significantly greater (30 minutes: 18.5 ± 1.4, 33.5 ± 1.4 and 53.9 ± 1.4%; 24 hours: 16.4 ± 0.9, 31.8 ± 1.0 and 48.3 ± 2.3% relative to the intact side at the dose of 30, 100 and 250 mg/kg, respectively) than those receiving the saline (30 minutes: 12.8 ± 1.6, 24 hours: 15 ± 0.8%, Repeated Measures of ANOVA, F_3, 16 _= 324, p < 0.0001 and posthoc t-tests of p's < 0.01, Fig. 6). DA fibers in the striatum of rats receiving edaravone at 30 minutes after 6-OHDA lesion was significantly preserved, compared to those with edaravone-administration at 24 hours (p's < 0.001), although DA neurons in the SNc of both time periods were not significantly different (p's = 0.11).

### TUNEL and HEt staining for anti-apoptotic and anti-oxidative effects

**Figure 7:**
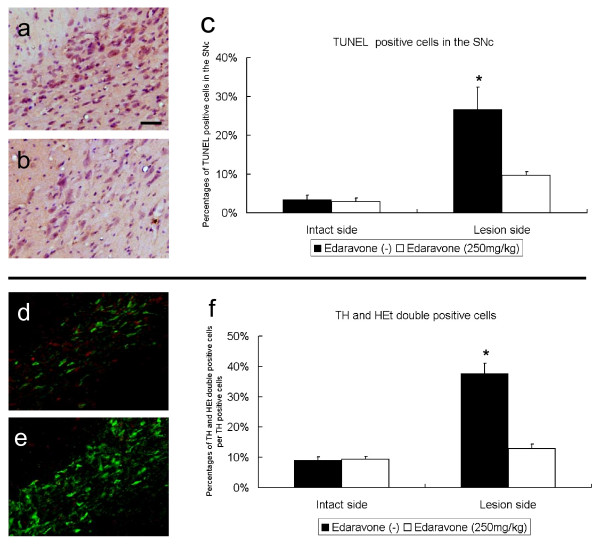
**Anti-apoptotic and anti-oxidative effects of edaravone**. Upper column: TUNEL staining revealed that edaravone administration (250 mg/kg) at 30 minutes after 6-OHDA lesion significantly decreased the percentages of TUNEL positive cells in the SNc (b), compared to rats without edaravone-administration (a). Scale bar: 60 μm The graph demonstrates the significant differences (c). Data are shown as the percentages of TUNEL positive cells +S.E. *p < 0.05 vs. the lesion side of edaravone-administered rats and the intact side. Lower column: TH and HEt double staining revealed that edaravone administration (250 mg/kg) at 30 minutes after 6-OHDA lesion significantly decreased the percentages of TH and HEt double positive cells (e), compared to rats without edaravone-administration (d; green: TH, red: HEt; Scale bar: 60 μm). The graph demonstrates the significant differences (f). Data are shown as the percentages of HEt positive cells +S.E. *p < 0.05 vs. the lesion side of edaravone-administered rats and the intact side.

The percentages of TUNEL positive cells in the SNc of rats receiving 250 mg/kg of edaravone at 30 minutes after 6-OHDA lesion significantly decreased (9.6 ± 1.0%), compared to those of rats without edaravone treatment (26.7 ± 5.7%, Repeated Measures of ANOVA, F_1, 12 _= 3.4, p = 0.034 and posthoc t-tests of p's < 0.05, Fig. 7). The percentages of TH and HEt double positive cells per TH positive cells of rats receiving 250 mg/kg of edaravone at 30 minutes after 6-OHDA lesion significantly decreased (12.9 ± 1.5%), compared to those of rats without edaravone treatment (37.7 ± 3.4%, Repeated Measures of ANOVA, F_1, 12 _= 32, p < 0.001 and posthoc t-tests of p's < 0.05, Fig. 7).

### Iba-1 immunohistochemistry for the affected inflammation

**Figure 8:**
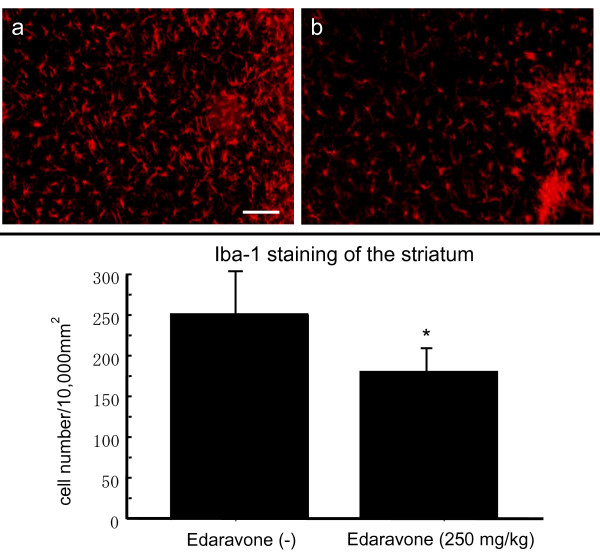
**Anti-inflammatory effects of edaravone**. Upper column: Iba-1 staining revealed that edaravone administration (250 mg/kg) at 30 minutes after 6-OHDA lesion significantly suppressed the number of Iba-1-positive cells (b), compared to rats without edaravone-administration (a), thus indicating anti-inflammatory effects of edaravone. Scale bar: 60 μm. Lower column: The graph demonstrates that 250 mg/kg of edaravone significantly suppressed the microglial proliferation, compared to rats without edaravone-administration. Data are shown as the cell number of Iba-1-positive cells +S.E. *p < 0.05 vs. rats without edaravone administration.

Iba-1 staining was performed to evaluate anti-inflammatory effects of edaravone through the microglia. Edaravone administration (250 mg/kg) at 30 minutes after 6-OHDA lesion significantly suppressed the number of Iba-1-positive cells (178 ± 4.2 cells/10,000 μm^2^; non-edaravone-administered group: 252 ± 14 cells/10,000 μm^2^), thus indicating that edaravone suppressed inflammation induced by 6-OHDA with the decrease of activated microglia (p < 0.05, Mann-Whitney's U test, Fig. 8).

## Discussion

In this study, neuroprotective effects of edaravone on 6-OHDA-treated murine ventral mesencephalic DA neurons were clarified *in vitro*. Anti-apoptotic effects through scavenging radicals might play an important role in the underlying mechanisms of neuroprotective effects of edaravone. In parallel, neuroprotective effects of edaravone on 6-OHDA-lesioned PD model of rats were demonstrated behaviorally and immunohistochemically. Edaravone might exert the neuroprotective effects on DA neurons. TUNEL, HEt and Iba-1 staining suggested the involvement of anti-apoptotic, anti-oxidative and anti-inflammatory effects of edaravone.

### Anti-apoptotic effects of edaravone

Neuroprotective effects of edaravone might be mediated by anti-apoptotic effects. Against ischemic reperfusion, edaravone prevents cell death and the release of cytochrome c with subsequent pathological apoptosis through Bcl-2 upregulation by inhibiting the opening of the mitochondrial permeability transposition pore [20,21]. In parallel, edaravone might reduce Fas-associated death domain protein and subsequently suppress apoptotic cell death in cerebral infarct [22]. Furthermore, edaravone might alleviate dysfunction of endoplasmic reticulum with subsequent cell death in cerebral ischemia [23]. Edaravone also reduces nitric oxide-induced apoptosis by inhibiting activation of MAP kinase in astroctes [24]. Related to 6-OHDA-toxicity, apoptosis is induced by down-regulation of Bcl-2 with activation of caspases in thymocytes [25], which might be suppressed by edaravone. Activated microglia damages surrounding cells by the paracrine of various cytokines. Using co-culture of neuronal cells and microglia, neuronal cell death by the peroxynitrite donor, SIN-1 (N-morpholinosydnonimine) is significantly suppressed by 10^-4 ^M edaravone [15]. In our study, the number of TUNEL-positive apoptotic cells and HEt-positive cells decreased using both *in vitro *and *in vivo *model of PD. The number of activated microglia of rats receiving 250 mg/kg of edaravone decreased, suggesting that the reduced cytotoxic cytokines might suppress apoptosis synergistically. The underlying mechanisms of the neuroprotection of edaravone might be involved in the hypothesis above described.

### Characteristics of our study

Recently, the similar study was reported using 1-methyl-4-phenyl-1,2,3,6-tetrahydropyridine (MPTP)-treated mice [26]. MPTP activated microglial activation both in the striatum and SNc with the increase of 3-nitrotyrosine, a biomarker of peroxynitrite production, in the SNc, but not in the striatum. Intraperitoneal 3 mg/kg of edaravone significantly ameliorate the behavioral scores, however the neuroprotective effects might be limited in the SNc. Using the animal model of cerebral infarct and head trauma, Dohi and colleagues also demonstrated the neuroprotective effects of low dose of edaravone [13]. In our study, the neuroprotective effects of edaravone (100 and 250 mg/kg) was demonstrated both in the striatum and SNc, although 30 mg/kg did not exert any neuroprotective effects, except for the histological amelioration in the striatum. Additionally, the behavioral amelioration by 100 mg/kg of edaravone-administered at 30 minutes and 24 hours after 6-OHDA lesioning did not show time-dependency. Furthermore, the lower dosage of edaravone (3 and 10 mg/kg) exerted no neuroprotective effects in our pilot study (data not shown). These discrepancies of the results might be due to the alteration of edaravone-activity and affinity over time after lesioning, the characteristics of the behavioral test, or the differences of the toxin (MPTP vs. 6-OHDA), of the administration route (i.p. vs. i.v.), of the animal species (mice vs. rat), and of the detailed regimen. For the safe clinical application, some amelioration of the drug, including the enhanced action for neurons specifically, because edaravone-administration even at clinical dosage might result in severe side effects [27].

Until now several studies demonstrated neuroprotective effects of pre-treatment of edaravone against metamphetamine-toxicity on striatal dopaminergic degeneration [28] and post-ischemic dopaminergic dysfunctions [29]. One of the remarkable characteristics of our study also lie in the time-dependent effects of edaravone, that is, the earlier (at 30 minutes after 6-OHDA lesion) administration might exert significantly stronger neuroprotective effects than the later one (at 24 hours), mimicking the clinical settings, although the later administration still displayed the behavioral and histological amelioration. In the future, the effects of repeated administration of edaravone on PD model should be clarified.

### Therapy for PD in the future including edaravone-administration

The established therapy for PD is medication using L-DOPA (dihydroxyphenylalanine), DA agonist and various drugs of different mechanisms, surgeries including electrical stimulation and ablation [30]. Fetal cell transplantation and GDNF infusion [31] are also hopeful, although the recent double-blinded randomized controlled trials questioned us the efficacy of these therapy [32,33]. In the nearest preceding years, neural transplantation might be a hopeful therapeutic option for PD [34] with recent development in the stem cell biology [35-40]. When edaravone is used for PD patients, several advantages might be recognized in combination with other therapeutic options. As edaravone extends the therapeutic time window for ischemic patients in combination with tissue plasminogen activator [41], edaravone might ameliorate the survival of transplanted cells [42] as well as scavenge free radicals in PD. Edaravone might also suppress inflammatory reaction induced by surgical procedures including electrical stimulation and cell transplantation.

## Conclusion

Neuroprotective effects of edaravone on 6-OHDA-treated DA neurons were clarified *in vitro*. Anti-apoptotic effects and radical scavenging activity might be involved in the underlying mechanisms of neuroprotective effects of edaravone. Neuroprotective effects of edaravone were then demonstrated using animal model of PD. Edaravone might be a hopeful therapeutic option for PD, although several critical issues remain to be solved, including high therapeutic dosage of edaravone for the safe clinical application in the future.

## Authors' contributions

WJY is involved in acquisition of data and drafting the manuscript. TS, TY, TA and ID designed the study, analyzed the data and revised the manuscript. KM, MK and YM performed *in vivo *experiments including surgeries and animal care. TU, TM and MJ performed in vitro experiments including immunocytochemical investigations. NT and TB performed immunohistochemical investigations. FW and LH performed additional experiments in the revised manuscript. All authors read and approved the final manuscript.
